# Rapid scaling up of screening for Sudan virus disease in healthcare facilities during the 2022 outbreak in Uganda

**DOI:** 10.1016/j.ijregi.2026.100983

**Published:** 2026-08-22

**Authors:** Shillah Nakato, Martin Esagala, Andrew Kwiringira, Judith Nanyondo, Abdullah Wailagala, Maureen Kesande, Doreen Nabawanuka, Paul Katongole, Isabella Fabens, Juliet Namugga Kasule, Dathan Mirembe Byonanebye, Francis Kakooza, Cori Dennison, Lisa Paige Oakley, Janelle Franklin, Elizabeth Bancroft, Ronny Reginald Bahatungire, Carolyn Herzig, Mohammed Lamorde

**Affiliations:** 1 Department of Global Health Security, Infectious Diseases Institute, College of Health Sciences, Makerere University, Kampala, Uganda; 2 World Health Organization (WHO), Uganda Country Office, Kampala, Uganda; 3 Makerere University, College of Health Sciences, Kampala, Uganda; 4 Centers for Disease Control and Prevention (CDC), Atlanta, Georgia; 5 School of Public Health, Makerere University, Kampala, Uganda; 6 The Ministry of Health (MoH), Kampala, Uganda; 7 Uganda Christian University, Mukono, Uganda

**Keywords:** Infection prevention and control (IPC), Sudan virus disease, Healthcare facility, Screening, Uganda, Outbreak

## Abstract

**Objectives:**

To rapidly address the 2022 Sudan virus disease (SVD) outbreak in Uganda, the Ministry of Health (MoH) and its partners initiated a multimodal infection prevention and control (IPC) strategy. We describe the strategy deployment in five high-risk districts and its effect on SVD screening capacity.

**Methods:**

The multimodal IPC strategy included rapidly distributing communication materials and IPC supplies to healthcare facilities (HCFs), training IPC mentors and supervisors, improving healthcare worker (HCW) knowledge through cascaded training and mentorship, and establishing screening at HCFs. IPC assessments focusing on screening capacity were conducted using the MoH IPC scorecard assessment tool, and changes in scores were compared across primary-, secondary-, and tertiary-level HCFs from baseline to endline.

**Results:**

IPC materials and supplies were distributed to 1135 HCFs, 296 IPC mentors were trained, and mentorship was cascaded to 3899 frontline HCWs. Of the 1135 HCFs assessed, 426 were analyzed. The mean screening capacity assessment scores increased significantly from baseline (44%) to endline (67%) in primary and secondary HCFs, especially in Health Center (HC) II (34-60%, *P* < 0.01), HC III (53-75%, *P* < 0.01), and HC IV (61-76%, *P* = 0.02). At the tertiary level, general and regional referral hospitals had higher baseline scores than HCFs at other levels and improved during these assessments (increases of 6 and 24 percentage points, respectively, between baseline and endline); however, these improvements were not statistically significant.

**Conclusion:**

The implementation of a multimodal IPC strategy rapidly enhanced the screening capacity of Uganda’s primary and secondary HCFs. However, tertiary HCFs showed little improvement. This strategy can be adopted for future outbreaks.

## Highlights

• This study describes screening as an initial action at health facilities that enhances early detection and source control.
• Screening improved healthcare facilities preparedness following the rapid implementation of the infection prevention and control intervention, with mean scores ranging from 34-60% in health center (HC) IIs, 53-75% in HC IIIs, and 61-76% in HC IVs.
• Screening capacity improved across all five assessed indicators, especially the availability of functional screening stations.
• The improvements at tertiary facilities were not significant, likely due to receipt of regular interventions.
• Significant improvements were noted in government, private for-profit, and private not-for-profit facilities across the implementation period.

## Introduction

Considered one of the administrative control methods based on the hierarchy of controls, screening is an effective way to prevent and control the spread of healthcare-associated infections, including Ebola virus disease (EVD) [1,2]. According to the World Health Organization (WHO), screening for Sudan virus disease (SVD) is the process of evaluating all patients, visitors, and healthcare workers (HCWs) at healthcare facility (HCF) entry points to identify individuals with signs and symptoms of SVD using a standardized tool and an approved case definition to ensure early detection, isolation, and prevention of transmission within the HCF [3]. This involves measuring the temperatures of all individuals at their first contact with the HCF to determine whether they meet the case definition using an infrared thermometer (no-touch) [1]. Previous Ebola outbreaks, such as the SVD in 2000, revealed how breaches in effective and consistent screening and other key infection prevention and control (IPC) practices led to infections among healthcare workers and patients [4,5]. Healthcare-associated infections lead to the closure of HCFs, disrupting the continuity of essential healthcare services [1].

Uganda's healthcare system is tiered, with the first tier comprising community-based Village Health Teams that provide basic health education, preventive care, and referrals to HCFs. The second tier, ranging from Health Center (HC) IIs and IIIs (primary-level HCFs) offering outpatient services and limited inpatient services (HC IIIs only) to HC IVs (secondary-level HCFs) at the county level, acts as mini-hospitals providing surgical services. In the tertiary tier, general hospitals provide more advanced care, regional referral hospitals (RRHs) offer specialized services and training, and national referral hospitals deliver highly specialized care. The system is complemented by private and faith-based organizations at all tiers. Due to their accessibility, patients typically visit primary or secondary HCFs before they present to tertiary-level HCFs. Implementing proper screening at the primary and secondary levels of HCFs is critical for the early detection, isolation, and referral of viral hemorrhagic fever cases, such as EVD or SVD, to minimize onward transmission within the healthcare system and the community.

On September 20, 2022, Uganda’s Ministry of Health (MoH) declared an SVD outbreak after confirming a case at Mubende Hospital, a rural RRH [5]. The outbreak spread rapidly to eight additional districts (Figure 1). The underlying factors that contributed to the rapid spread included uncontrolled cross-district movements, breaches in IPC practices, cultural practices such as washing the bodies of deceased persons, large gatherings during funerals at the start of the outbreak, and the use of traditional medicines [5,6]. The MoH, in collaboration with the Infectious Diseases Institute (IDI) of Makerere University and partners such as WHO and the US Centers for Disease Control and Prevention (CDC), implemented various response measures to control the outbreak [7]. Control measures included strengthening disease surveillance, contact tracing, and a multimodal IPC strategy. We describe the rapid implementation of a multimodal IPC strategy and its effect on screening capacity at HCFs in five of the nine districts in Uganda that reported cases during the 2022 SVD outbreak (Figure 1).

**Figure 1:**
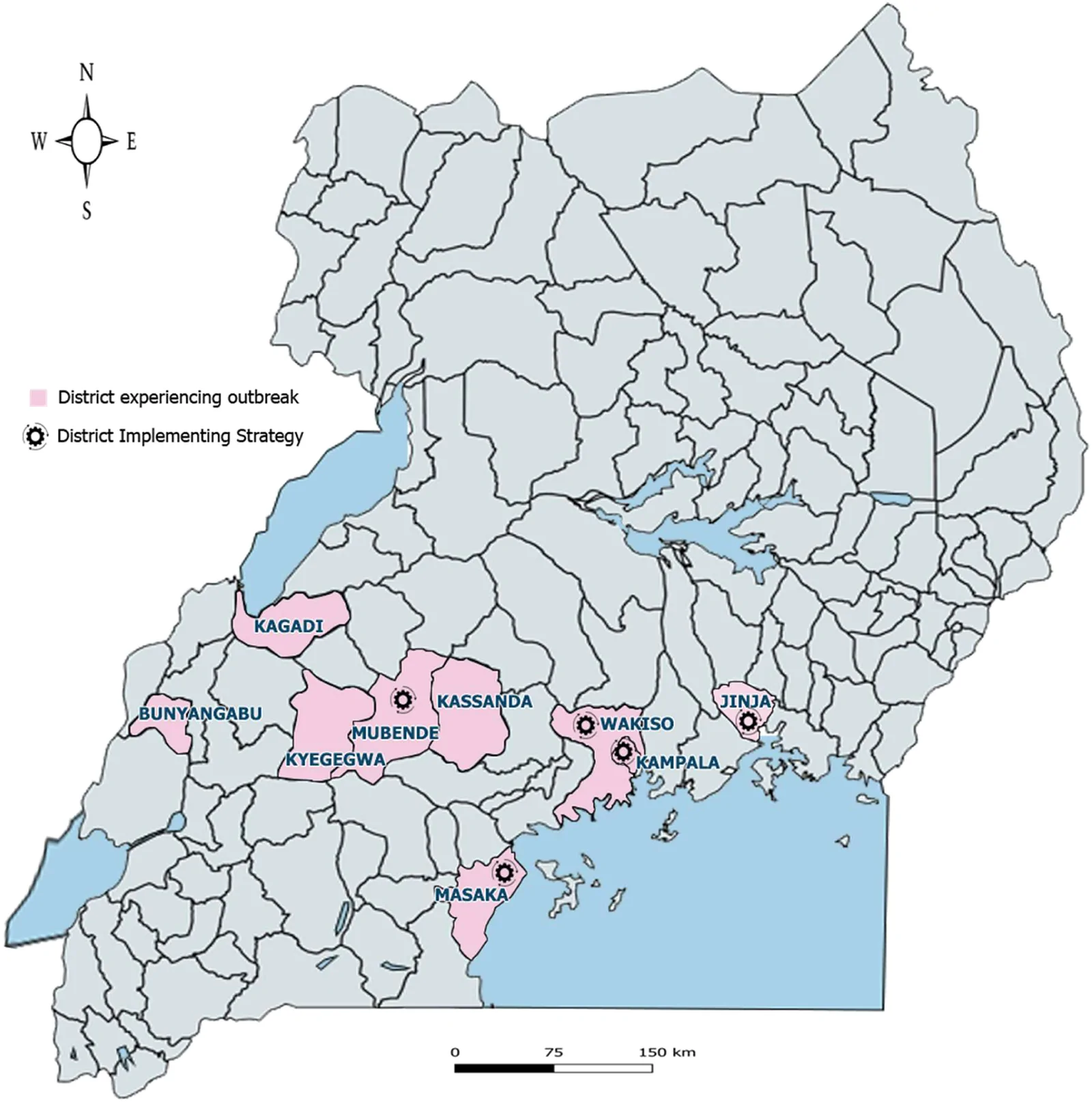
Map of Uganda showing the 2022 Sudan virus disease (SVD) outbreak districts, including those that implemented the strategy.

## Methods

We rapidly deployed a multimodal IPC strategy in 1135 healthcare facilities across five high-risk districts affected by the 2022 SVD outbreak between November 2022 and January 2023.

Interventions in the multimodal IPC strategy included distributing information, education, and communication (IEC) materials and IPC supplies; improving the skills and knowledge of HCWs through onsite training and mentorship; and setting up and functionalizing screening at HCFs. These interventions were selected based on HCF IPC assessments to identify existing gaps.

### Rapid distribution of IEC materials to high-risk districts

IEC materials outlining case definitions were printed and distributed to HCFs and proximal community settings such as markets, schools, and churches in high-risk districts. Case definitions were developed at the start of the outbreak by the surveillance pillar under the Uganda MoH Incidence Management System. This activity was conducted to rapidly increase HCW and public awareness of SVD signs and symptoms and to increase the reporting of suspected cases. To improve screening and hand hygiene practices, we also distributed screening algorithms and posters to HCFs in the affected districts on hand hygiene (e.g., “My 5 moments of hand hygiene” [8]), how to properly wash hands with soap or use alcohol-based hand rub, how to use an infrared (no-touch) thermometer, and MoH-approved standard operating procedures (SOPs) on screening visitors, patients, and HCWs.

### Training of IPC mentors and HCWs in high-risk districts

With support from the district health leadership, district IPC mentors were selected from among HCWs within the districts. The selection criteria included having prior knowledge of IPC, being a previous mentor or an IPC focal person at an HCF, the capacity to operate a smartphone, and the attributes of a good trainer (confidence, good communication and interpersonal skills, knowledge, and patience). IDI, in collaboration with the MoH and CDC, coordinated regional training for the selected mentors. The 3-day didactic and practical training was specific to the HCF setting and included the following topics: personal protective equipment use, HCF screening (patients upon entry, among HCWs, and among inpatients at secondary-level HCFs), isolation and notification, environmental cleaning, management of linens and patient equipment, basics of SVD transmission, and conducting MoH standardized IPC scorecard assessments. The mentors completed pre- and post-tests to evaluate the knowledge gained during the training. Only those who passed the post-test with a score of at least 70% were involved in HCF visits to provide mentorship to HCWs within their districts. These mentors were tasked with cascading this training to frontline HCWs at the assigned HCFs and reinforcing their knowledge through ongoing in-person HCF mentorship visits.

### IPC assessments

Trained IPC mentors used the MoH IPC scorecard assessment tool for EVD [9], adopted from the WHO IPC Rapid Assessment scorecard for EVD, to conduct baseline assessments and collect data on 15 capacity domains during every HCF visit. Mentors focused on five prioritized screening capacity indicators: (1) the HCF has a screening station at each entry point, (2) there is a distance of at least 1 m between the patient/visitor and the screener, (3) a functional thermoflash (infrared thermometer) is available at every screening point, (4) temperature is correctly measured and verified at screening points, and (5) suspected cases are referred to holding/isolation areas.

### Mentorship and supervisory visits to improve health facility screening capacity

Every trained IPC mentor received a mentorship package with SOPs that served as reference materials during visits. The mentors were initially assigned to visit two HCFs once a week for 1 month. During this time, they worked with the IPC committees at each HCF to conduct baseline assessments using the MoH IPC scorecard assessment tool for all IPC capacity domains, with a focus on screening. After 1 month, each IPC mentor was assigned additional HCFs to support for 1 month, influenced by the dynamic outbreak context. As the outbreak evolved, priorities and operational needs changed, requiring flexibility in the allocation and focus on low performing HCFs. The HCF IPC assessment results were used to guide ongoing interventions and determine the frequency of ongoing in-person mentorship. To ensure the fidelity of intervention delivery, IPC mentors were supported by IPC supervisors selected from among graduate nurses, doctors, and public health professionals at the national level, who had supported previous outbreak responses and were considered to have extensive IPC training and experience. IPC supervisors were assigned to specific geographic areas within the five high-risk districts and visited the three HCFs weekly. At the HCFs, they reviewed the IPC assessment results with the HCF staff, mentored HCWs on all IPC capacity domains of the IPC scorecard assessment tool, and provided the support needed by mentors. IPC supervisors visited HCFs based on the overall results of the assessments conducted by IPC mentors. Any HCF with an overall IPC capacity score below 50% was prioritized to continue receiving mentorship from either the IPC mentor or supervisor for an additional 1 month during the outbreak period and through the 6-month post-outbreak recovery period.

### Setting up and functionalizing screening at HCFs

Mentors worked with HCWs to establish screening stations and functionalize screening at HCF entry points. A screening area was considered functional if the HCF responded “yes” to all five screening capacity indicators during the assessment; any “no” response was deemed the screening area “non-functional.” Continuous medical education sessions and onsite mentorships were conducted to enhance HCW knowledge of screening practices. Additionally, mentors observed HCWs while screening people entering HCFs and conducted one-on-one mentorship sessions when gaps were identified. Text message reminders, SOPs, and communication materials were provided to enhance the HCWs’ knowledge of the screening process. Additionally, IPC supplies, including infrared thermometers, were distributed to the HCFs to functionalize the screening stations.

### Data collection

The MoH IPC scorecard assessment tool [9] was digitized using the Open Data Kit [10], an open-source data collection platform. The Open Data Kit was installed on the mentors’ smartphones for IPC assessment data collection. This tool was hosted on the MoH server. All collected data were archived on the MoH server. Access to the server was restricted to authorized members of the MoH IPC coordination team. For analysis, the data were extracted from the server in comma-separated value format and imported into statistical software for data cleaning and analysis.

### Data management and analysis

Data quality was maintained through several measures embedded within the electronic data collection system, including the use of a standardized digital MoH IPC assessment tool, predefined response options, mandatory fields, and routine review of submitted assessments by IPC supervisors during implementation. In addition, data cleaning was performed before analysis to identify and resolve inconsistencies and incomplete records.

Only HCFs with at least two assessments were included in the analysis. Descriptive statistics were calculated, and significant differences were determined using paired *t*-tests and chi-square tests for continuous and categorical variables, respectively. We compared the mean scores for the screening capacity indicators at baseline and endline within each category of ownership (government vs private for-profit vs private not-for-profit) and within each HCF level. SPSS Statistics software (version 20.0; IBM Corp., Armonk, NY, USA) was used for the analysis.

## Results

### Demographics

Assessment data from 1135 HCFs within five high-risk districts were collected between November 2022 and January 2023. Due to staffing shortages, only 426 (37.5%) HCFs underwent at least two assessments (baseline and endline), and these data were analyzed. Of the 426 HCFs, 23% (*n* = 99) were government facilities, 62% (*n* = 263) were private for-profit facilities, and 15% (*n* = 64) were private not-for-profit facilities. More than half of the HCFs (57%, *n* = 244) were HC IIs; 26% (*n* = 111) were HC IIIs; 11% (*n* = 45) were HC IVs; 4% (*n* = 19) were hospitals; 1% (*n* = 5) were RRHs and 0.5% (n=2) were NRHs (Table 1).

**Table 1:** Mean screening assessment scores for Sudan virus disease at healthcare facilities in Uganda at baseline and endline, November 2022-January 2023.

	Mean at baseline (%)	Mean at endline (%)	Absolute difference between mean endline and baseline scores (%)	*P*-value
**Level of health facility**				
HC II (*n* = 244)	34	60	26	<0.01
HC III (*n* = 111)	53	75	22	<0.01
HC IV (*n* = 45)	61	76	15	0.02
Hospital (*n* = 19)	67	73	6	0.49
RRH (*n* = 5)	72	96	24	0.50
NRH *(n = 2)*	60	60	0	1.0
**Ownership**				
Government (*n* = 99)	54	76	22	<0.01
PFP (*n* = 263)	35	62	27	<0.01
PNFP (*n* = 64)	62	73	11	0.02

Abbreviations: HC, health center; PFP, private for-profit; PNFP, private not-for-profit; RRH, regional referral hospital; NRH, national referral hospital.

### Distribution of IEC and IPC materials and training of HCWs

We distributed 10,000 case definition posters, 1,800 infrared thermometers, and 3,200 l of alcohol-based hand rub to 1135 HCFs and proximal community settings, including markets, schools, and churches, in five high-risk districts. We trained 296 IPC mentors who cascaded mentorship to 3899 HCWs.

### Screening capacity assessment scores

Baseline screening assessment scores varied among the primary, secondary, and tertiary levels of care, with primary care HCFs presenting the lowest baseline scores. Significant improvements in screening were observed between baseline and endline in HC II (34-60%, *P* < 0.01), HC III (53-75%, *P* < 0.01), and HC IV (61-76%, *P* = 0.02). While improvements were also observed in hospitals and RRHs (increases of 6 and 24 percentage points between baseline and endline, respectively), these tertiary-level HCFs already had higher scores at baseline because of previous significant IPC support; thus, the change between baseline and endline was not statistically significant (Table 1). Endline screening assessment scores were significantly higher than at baseline for all categories of ownership: 76% vs 54% (*P* < 0.01), 62% vs 35% (*P* < 0.01), and 73% vs 62% (*P* = 0.02) for government, private for-profit, and private not-for-profit healthcare facilities, respectively (Table 1).

The mean scores for each of the five screening capacity indicators increased for all facilities, and the indicator with the greatest improvement (absolute increase of 26 percentage points) was the presence of a functional screening station at each entry point into the HCF (Figure 2). At baseline, 20% (*n* = 83) of the healthcare facilities had functional screening areas (i.e., responded “yes” to all five screening capacity indicators). This increased significantly to 59% (*n* = 251) by the end of the intervention period (*P* < 0.01). By the end of the outbreak, following the implementation of the interventions, 16 of 251 (6%) supported healthcare facilities with a functional screening system reported suspected SVD cases during routine IPC assessments, in accordance with national screening and reporting procedures.

**Figure 2:**
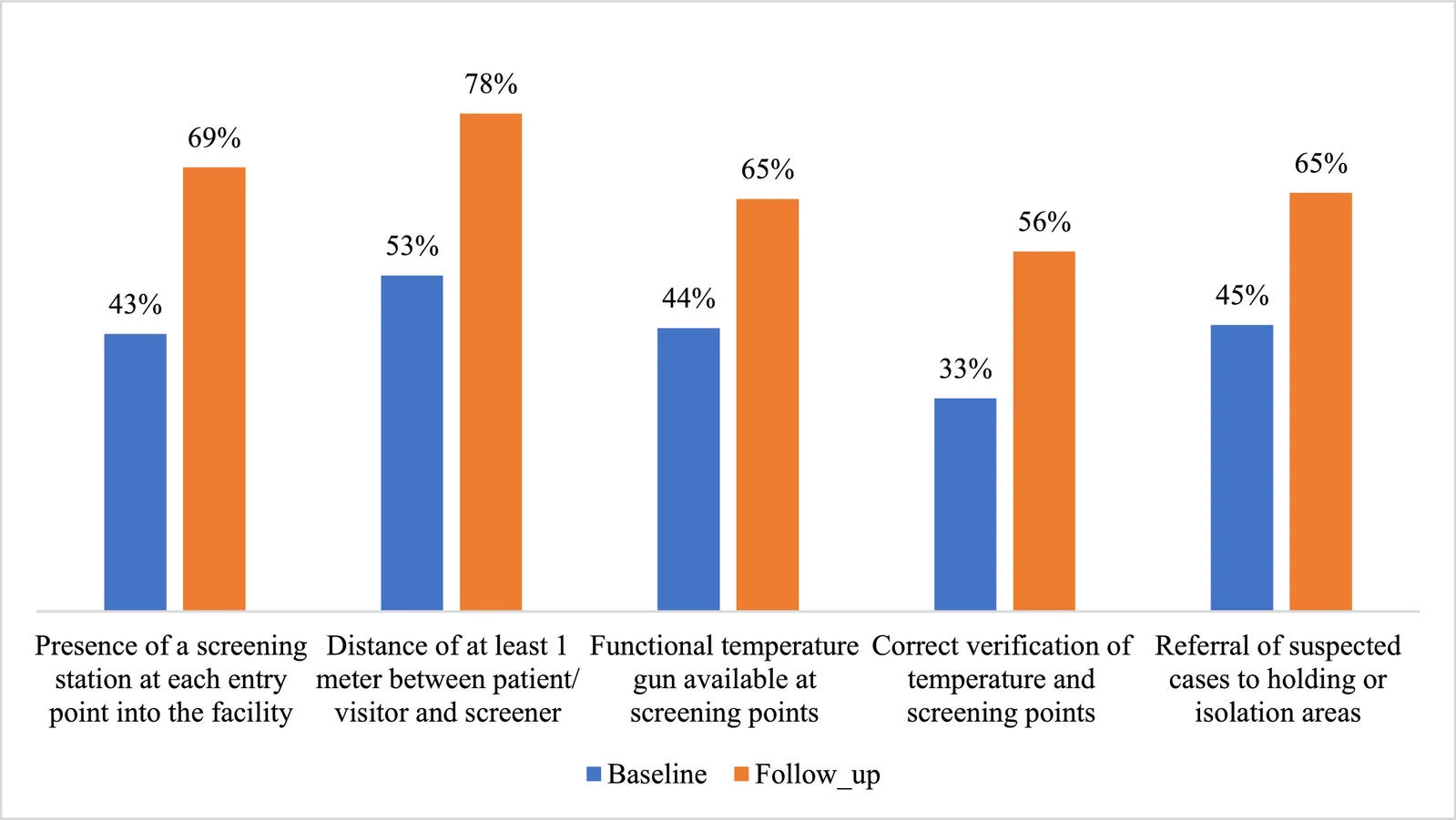
Mean baseline and endline scores for each screening parameter.

## Discussion

We used multimodal IPC strategies to improve HCF screening during an active SVD outbreak in Uganda. This was accomplished through the distribution of IEC materials and essential IPC supplies, continuous monitoring and feedback, leadership engagement, onsite mentorship and training to improve skills and knowledge among HCWs, and the establishment of functional screening stations. These interventions acted synergistically to improve screening through the interaction of multiple strategies rather than through a single component, with strong collaboration between the MoH and partners.

The proportion of primary- and secondary-level HCFs with functional screening areas increased significantly by the end of the intervention, with tertiary-level HCFs showing slight improvement in their already high screening practices. This clearly demonstrates the impact of the targeted support and capacity-building efforts. This underscores the importance of structured IPC interventions to rapidly enhance HCF readiness during outbreaks and control infections at the source through screening. As a result of the interventions, only 6% (16/251) of the supported HCFs with functional screening reported suspected SVD cases post-implementation. Early identification and notification of suspected SVD cases facilitate timely management to improve patient outcomes and prevent exposure to other patients and HCWs. Despite the relatively low number of HCFs reporting suspected SVD cases, even with enhanced screening measures, improving the infrastructure alone is insufficient. Gaps in staffing, training, or reporting mechanisms could have contributed to the continued reporting of suspected cases, indicating the need for ongoing support and staffing to ensure effective case detection [11]. Overall, these findings underscore the need for strategies that combine facility readiness with continuous capacity building and monitoring to improve disease identification and outbreak response [12].

Secondary- and tertiary-level healthcare facilities demonstrated higher baseline screening assessment scores than did primary-level facilities. This likely reflects prior interventions in IPC capacity, particularly during the COVID-19 pandemic when many secondary- and tertiary-level facilities served as designated COVID-19 treatment centers and received enhanced IPC support, including training, mentorship, infrastructure improvements, and essential IPC supplies. These cumulative interventions likely resulted in more established IPC programs and higher baseline screening performance in secondary- and tertiary-level healthcare facilities than in primary healthcare facilities [[12], [13], [14], [15]]. However, this outbreak highlights the importance of strengthening IPC programs in primary- and secondary-level HCFs, which serve as the first point of contact with the healthcare system for many patients, including those with viral hemorrhagic fever such as EVD. Improving IPC capacity at primary- and secondary-level HCFs has been shown to reduce healthcare-associated transmission and control outbreaks [11,[16], [17], [18]]. A multimodal implementation approach comprising assessment-guided mentorship and the provision of essential IPC supplies strengthens the screening capacity across supported healthcare facilities [19,20].

The involvement of district health leadership was critical in identifying and selecting IPC mentors, supervising HCFs, accessing the necessary IPC supplies, and promoting adherence to screening protocols [21]. District health leadership support led to greater facility engagement and improved HCW uptake of IPC practices through in-person facility visits and support in addressing IPC gaps, facilitated by transportation reimbursement. This engagement aligns with prior findings underscoring the importance of decentralized response strategies [17].

The outbreak also demonstrates the need to strengthen IPC measures in private healthcare facilities. Although public HCFs had previously been prioritized during the COVID-19 response [14,15], private HCFs continue to play a central role in Uganda’s healthcare system, delivering approximately 60% of all healthcare services in the country and serving as the first point of contact for up to 70% of the population [22]. Therefore, integrating private facilities into national IPC frameworks is crucial for comprehensive outbreak preparedness and response.

### Strengths and limitations

The multimodal strategy described here had some limitations: (i) the intervention primarily aimed to rapidly reduce disease transmission rather than strengthen the IPC foundation of the Ugandan health system in a long-term, sustainable manner. Consequently, several HCFs lacked the resources necessary to sustain key IPC interventions during the recovery period. Focusing on the most immediate outbreak needs was necessary in this case; however, linking the IPC gains made between response and non-response periods is critical for the long-term success of IPC programs at HCFs; (ii) while the screening assessment effectively identified the presence of screening structures, only five screening capacity indicators out of six from the WHO IPC rapid scorecard assessment for EVD were included in the Uganda scorecard assessment, providing an incomplete picture of screening functionality and quality at HCFs (the component on availability of a questionnaire or screening tool based on the national case definition was not assessed); (iii) the analysis was restricted to 426 HCFs that had at least two IPC assessments, representing only 37.5% of the total healthcare facilities included in the intervention. This could have resulted in selection bias, as facilities that did not receive follow-up assessments may have differed systematically in capacity or performance. Severe staffing shortages limited the number of facilities that had an endline assessment conducted; (iv) these findings may not be generalizable beyond the HCFs in the five high-risk districts where data were collected or to other settings; (v) there was an unequal distribution of healthcare facilities across levels; primary-level healthcare facilities were the majority, while secondary- and tertiary-level facilities were comparatively fewer. However, this mirrors the national health system rather than the implementation design. This imbalance may have influenced the overall findings and limited the precision of comparisons across HCF levels, particularly for secondary- and tertiary-level facilities with smaller sample sizes; and (vi) we also acknowledge the absence of baseline data on reported cases during screening at HCFs as a limitation and note that future implementation evaluations designed as research studies should incorporate baseline and follow-up measures to more robustly evaluate the effect of screening interventions on case detection and reporting.

To build a robust health system capable of managing future infectious disease threats, sustainable IPC interventions must be institutionalized across all levels of care. This includes embedding IPC training in both pre-service and in-service education, establishing continuous mentorship and support programs, and ensuring routine monitoring and evaluation mechanisms are in place [23]. Such investments are necessary not only to sustain improvements achieved during outbreaks but also to enhance health system resilience more broadly. Addressing these limitations through ongoing support and strategic planning is essential to improve future outbreak preparedness and response.

## Conclusion

The implementation of a multimodal IPC strategy improved screening capacity across healthcare facilities during the 2022 SVD outbreak in Uganda. Screening functionality scores improved significantly within a relatively short implementation period, mostly among primary- and secondary-level public and private healthcare facilities. These findings suggest that coordinated implementation strategies combining mentorship, training, the provision of IPC supplies, and supportive supervision can strengthen IPC screening practices during outbreak responses. The identification and reporting of suspected cases in light of improved screening underscore the need to ensure that IPC practices are accompanied by effective disease detection and response.

Sustaining these gains requires continuous capacity building, integration of IPC into routine health-service delivery, and regular monitoring across all levels of the healthcare system. Addressing systemic challenges such as human resource constraints, supply constraints, and resource allocation limitations remains critical for strengthening health system resilience and improving preparedness for future outbreaks, including SVD.

## CRediT authorship contribution statement

**Shillah Nakato:** Conceptualization, Data curation, Formal analysis, Investigation, Methodology, Project administration, Resources, Software, Supervision, Validation, Visualization, Writing – original draft, Writing – review & editing. **Martin Esagala:** Conceptualization, Data curation, Formal analysis, Writing – original draft, Writing – review & editing. **Andrew Kwiringira:** Formal analysis, Project administration, Software, Writing – original draft, Writing – review & editing. **Judith Nanyondo:** Conceptualization, Project administration, Writing – review & editing, Funding acquisition, Methodology, Resources, Supervision. **Abdullah Wailagala:** Writing – original draft, Writing – review & editing. **Maureen Kesande:** Methodology, Writing – original draft, Writing – review & editing. **Doreen Nabawanuka:** Writing – review & editing. **Paul Katongole:** Funding acquisition, Methodology, Writing – review & editing. **Isabella Fabens:** Conceptualization, Methodology, Project administration, Writing – original draft, Writing – review & editing. **Juliet Namugga Kasule:** Funding acquisition, Writing – review & editing. **Dathan Mirembe Byonanebye:** Writing – review & editing. **Francis Kakooza:** Funding acquisition, Project administration. **Cori Dennison:** Writing – review & editing. **Lisa Paige Oakley:** Writing – review & editing. **Janelle Franklin:** Funding acquisition, Conceptualization, Data curation, Formal analysis, Methodology, Project administration, Resources, Software, Supervision, Validation, Visualization, Writing – review & editing. **Elizabeth Bancroft:** Funding acquisition. **Ronny Reginald Bahatungire:** Project administration. **Carolyn Herzig:** Funding acquisition, Data curation, Methodology, Project administration, Resources, Supervision, Writing – original draft, Writing – review & editing. **Mohammed Lamorde:** Funding acquisition, Project administration, Resources.

## Declaration of competing interest

The authors have no competing interests to declare.

## Appendix Supplementary materials

Image, application 1
