# Supplementary material for: Rapid scaling up of screening for Sudan virus disease in healthcare facilities during the 2022 outbreak in Uganda
Source: IJID Reg. 2026 Aug 22;21:100983. doi: 10.1016/j.ijregi.2026.100983 (PMC13627929; doi:10.1016/j.ijregi.2026.100983)
Supplement: Supplementary file 1 [file mmc1.pdf]

Telephone: General Lines: 256 – 417 – 712260
Permanent Secretary's Office: 256 – 417 – 712221
Toll Free: 0800100066
E-mail: ps@health.go.ug
Website: www.health.go.ug

FOR GOD AND MY COUNTRY
THE REPUBLIC OF UGANDA

Ministry of Health
P. O. Box 7272
Plot 6, Lourdel Road,
Wandegeya
KAMPALA
UGANDA

IN ANY CORRESPONDENCE ON.
THIS SUBJECT PLEASE QUOTE NO. ADM 017/551/01

20th March, 2022

To Whom it May Concern

RE: NO OBJECTION TO PUBLICATION AND DISSEMINATION OF STUDY FINDINGS

The Ministry of Health, National Task Force reviewed the manuscript "Rapid Scaling-Up of Screening for Sudan Ebola Disease in Healthcare Facilities During the 2022 Outbreak in Uganda" and has no objection to its publication or dissemination.

The activities described in the manuscript were conducted as part of routine public health emergency response activities. Data were collected as part of routine public health surveillance and program monitoring to support the emergency response. The analysis involved routinely collected programmatic data and did not constitute human subjects research under the Ministry of Health's outbreak response mandate.

This no-objection is granted on the understanding that:

1. The publication accurately reflects the findings of the work.
2. No confidential, classified, or personally identifiable information is disclosed.
3. The interpretations and conclusions presented are those of the authors and do not necessarily represent the official policy or position of the Ministry of Health.

The Ministry of Health appreciates the efforts of the authors in documenting Uganda's experience and lessons learned from the 2022 Sudan Ebola Disease outbreak response. Dissemination of these findings will contribute to strengthening the evidence base for preparedness and response to future public health emergencies.

Thank you

Dr. Charles Olaro

DIRECTOR GENERAL HEALTH SERVICES
